# Intravascular ultrasound wall shear stress imaging in stented coronary arteries with ultrafast Doppler

**DOI:** 10.1038/s41598-026-47719-9

**Published:** 2026-04-09

**Authors:** Travis C. Singh, Stephan Strassle Rojas, Imran Shah, Jimena Martín Tempestti, Alessandro Veneziani, Brooks D. Lindsey

**Affiliations:** 1 Wallace H. Coulter Department of Biomedical Engineering, Georgia Institute of Technology and Emory University, 313 Ferst Dr. NW, Atlanta, GA 30332 USA; 2 School of Electrical and Computer Engineering, Georgia Institute of Technology, 791 Atlantic Dr. NW, Atlanta, GA 30332 USA; 3 Department of Mathematics, Emory University, 301 Dowman Dr., Atlanta, GA 30322 USA; 4 Department of Computer Science, Emory University, 400 Dowman Dr., Atlanta, GA 30322 USA

**Keywords:** Wall shear stress, Intravascular ultrasound, Stent malapposition, Blood flow velocity, Coronary artery, Percutaneous coronary intervention optimization, Cardiology, Computational biology and bioinformatics, Diseases, Medical research

## Abstract

Percutaneous coronary intervention (PCI) is a common minimally-invasive procedure for treating coronary artery stenosis. However, 10% of patients with non-complex lesions experience restenosis within five years of initial PCI. Wall shear stress (WSS) is a physiological marker that provides additional predictive value for restenosis. The ability to estimate WSS during PCI could identify patients at high risk for restenosis. In order to assess the accuracy of intravascular ultrasound WSS imaging in coronary geometries, a high-frequency linear array was used to image three unique coronary phantom geometries before stenting and with partially- and fully-expanded stents. Acquired 2D WSS images were registered and compared with 3D in silico results. Finally, 3D images were acquired in a single geometry using a newly-developed intravascular ultrasound matrix array. Ultrasound-derived flow velocity maps demonstrated a mean absolute percentage error of 13.04 ± 4.82% relative to simulations, with a mean correlation of 81.91 ± 14.59%. Segments with partially-expanded stents exhibited decreased mean WSS (0.0800 ± 0.0233 Pa vs. 0.1328 ± 0.0265 Pa, *p* = 0.0479) and increased WSS spatial variance (0.0038 ± 0.0011 Pa^2^ vs. 0.00069 ± 0.000090 Pa^2^, *p* = 0.0546) compared to segments with fully expanded stents. Accurate WSS imaging during PCI could stratify restenosis risk and inform long-term coronary modeling (i.e. digital twin system).

**Supplementary Information:**

The online version contains supplementary material available at 10.1038/s41598-026-47719-9.

## Introduction

In 2019, coronary artery disease (CAD) affected 124 million individuals, was responsible for 9 million deaths worldwide, and was the leading contributor to disability-adjusted life years (DALYs) with 164 million years^1,2^. Atherosclerosis is the primary cause of CAD and is characterized by plaque accumulation within the coronary arteries. While atherosclerosis is highly multifactorial, it is generally understood to initiate with damage to the endothelium from oxidative stress, which leads to deposition of low-density lipoproteins and in turn promotes a positive feedback cycle of leukocyte recruitment, foam cell formation, necrosis and apoptosis, and smooth muscle proliferation^3,4^.

Symptomatic CAD-patients often experience chest pain due to ischemia. Percutaneous coronary intervention (PCI) is the preferred treatment for relieving symptoms of ischemia, with approximately 3 million procedures performed annually^5,6^. PCI is preferred to coronary artery bypass graft (CABG) due to its minimally invasive nature, decreased recovery times and mortality rates, and reduced risk for post-procedural stroke^7,8^. The introduction of drug-eluting stents (DES) further improved treatment of CAD, reducing restenosis rates to approximately 2–10% for non-complex lesions in the first year post-PCI^9^. While improvements from bare metal stents to DES have greatly improved patient outcomes, it remains that approximately 10% of patients with non-complex lesions still experience in-stent restenosis or stent thrombosis within 5 years of their initial PCI^10–14^. For complex lesions, the risk for restenosis increases to 13–20% over a 5-year period^15^. Additionally, 25%−33% of patients experience recurrent angina by the 1-year follow-up and/or a major adverse cardiac event (MACE) such as death, nonfatal myocardial infarction (MI), or target lesion revascularization by the 2-year follow-up^16^.

Current consensus guidelines recommend the use of intracoronary imaging—optical coherence tomography (OCT) or intravascular ultrasound (IVUS)—in addition to angiography to improve patient outcomes and reduce in-stent restenosis rates^10,17–19^. Despite these recommendations, angiography alone is used in 83.8% of all PCI cases^20^. Furthermore, angiography forms 2D projection images of 3D anatomy which, limits its ability to assess stent apposition, minimal luminal area (MLA), and plaque composition^21^. Evaluation of these criteria using angiography alone may result in stent failure due to underexpansion, edge dissection, or geographical miss, which are all associated with elevated risk for MACE and restenosis^12,21^.

Current intracoronary imaging techniques only provide geometric assessment in coronary arteries. Considering imaging modalities, IVUS can characterize the plaque and stent apposition with a spatial resolution of ~ 40 μm at 40 MHz and a penetration depth of ~ 6 mm^22^. When PCI is guided by IVUS, long-term cardiac death and revascularization rates are significantly reduced compared to angiography-guided PCI in complex lesions^12^. However, current IVUS devices lack the ability to image hemodynamics because they are side viewing, and thus the ultrasound beam is orthogonal to the primary direction of blood flow.

OCT provides higher spatial resolution (~ 10 μm), thus OCT can assess the plaque and geometric apposition, however, it lacks the ability to image local hemodynamics^23,24^. In addition to geometric apposition assessment, the ability to image hemodynamics could reveal underlying factors that may lead to restenosis in an individual patient to stratify risk of restenosis. For example, neoatherosclerosis or neointimal hyperplasia are hemodynamic-dependent causes of restenosis^25^. Hemodynamic characterization could influence lesion preparation, choice of stent type and size, deployment strategy, and post-implantation assessment of stent expansion and apposition, and risk of restenosis after stent deployment^26^.

Wall shear stress (WSS) is an established hemodynamic marker that refers to the tangential component of the normal stress acting along the boundary in an incompressible fluid^27,28^. WSS is correlated also an indicator of likelihood of restenosis^29–32^. Elevated WSS is associated with expansive vascular remodeling and increased plaque vulnerability^33–37^. In the PROSPECT trial, locally decreased WSS was found to provide incremental risk stratification for non-stented lesions in high-risk patients compared to plaque burden, MLA, and morphology alone^31^. Therefore, understanding the impact of stents on WSS at the target lesion is important for characterizing risk of restenosis post-PCI.

Post-PCI, stents have been shown to cause a variety of changes to the local hemodynamics. The oscillatory pattern of the struts along the vessel wall generates regions of high WSS at the struts and low WSS in the regions between the struts, disrupting the local hemodynamics^38^. Furthermore, underexpansion of stents is associated with high WSS^39^, while the stent footprint alone results in low local WSS^40^. Finally, malapposed struts induce significant changes such as transitions from laminar to disturbed flow and the generation of recirculation zones^41^. Thus, the ability to image these complex WSS interactions could allow interventionalists to further optimize stent placement during PCI and improve risk stratification for patients post-PCI.

In order to estimate and spatially map WSS, both anatomical and physiological measurements are required. The current standard for local WSS estimation is to use anatomical imaging modalities including computed tomography angiography (CTA) and IVUS or OCT with wire-based measurements (e.g., FFR) as inputs to computational fluid dynamic (CFD) modeling^42–44^. CFD in patient-specific geometries can be highly accurate if patient-specific measurements are acquired to provide the required boundary conditions, however, it is computationally expensive^45,46^. Several studies have utilized CFD to investigate the hemodynamic impact of stent footprints in idealized or animal artery models^47–50^. Recent investigations have embedded stents in patient-specific geometries and then simulated hemodynamics based on OCT^51–54^. These studies have shown that the presence of the stent struts leads to lower WSS and disturbed flow patterns^40,55^. For example, in a longitudinal study of 12 patients, a negative correlation was shown between WSS and plaque progression^55^. Related studies reported similar results in the absence of clear malapposition^56,57^. Thus, although CFD lacks the ability to estimate WSS in real-time for PCI optimization, it is the best current method for hemodynamic assessment and may offer incremental information to inform long-term clinical decision-making, including as part of a digital-twin system post-PCI.

As an alternative to CFD, imaging modalities could be augmented to map WSS and guide clinical decision-making. Currently, WSS in coronary arteries can be estimated with imaging using phase contrast MRI (PC-MRI) or ultrasound. PC-MRI is used to estimate WSS in vivo in larger vessels like carotid arteries and the aorta, however, it is challenging to accurately estimate WSS in small, mobile arteries like coronary arteries due to the spatial and temporal resolution requirements^58–61^. Alternatively, ultrasound (US) has been used to estimate WSS in vivo in larger arteries like the femoral, aorta, and carotid arteries^62,63^. Furthermore, development of ultrasound techniques like multi-angle vector flow imaging and ultrafast plane-wave imaging has provided 2D and 3D blood flow velocity mapping at even higher frame rates in the carotid bifurcation, brachial, and femoral arteries^64–66^.

These prior studies demonstrating US-based WSS estimation have utilized large, non-invasive arrays that lack the spatial resolution and penetration depth needed to accurately map WSS in coronary arteries. Given that intra-coronary imaging such as IVUS is recommended for PCI guidance, if forward-viewing IVUS (FV-IVUS) could simultaneously image anatomy and blood flow velocity, then WSS imaging—an additional marker with incremental value for risk stratification—could be provided to the interventional cardiologist during the PCI procedure. This would require a FV-IVUS matrix array, which we have been developing and evaluating in our lab^67,68^. If WSS variation in stented arteries can be accurately estimated with FV-IVUS, it may be possible to optimize stenting and identify high-risk patients for early follow-up (e.g., stress testing or angiography in 3–6 months) or adjunct therapy like cilostazol, per prior guidelines^69,70^. Finally, the anatomical and physiological data might also serve as inputs to a coronary health digital twin system to predict long-term patient outcomes^71,72^.

The goal of this work is to demonstrate that US imaging alone can accurately map WSS in coronary artery geometries pre- and post-stenting. We present the first study of US-based WSS imaging in stented coronary geometries. WSS maps were formed (1) in physical tissue-mimicking phantoms with US and (2) in silico in the same geometries via CFD. In addition to these 2D US images acquired with a commercial high-frequency, we also present as an initial proof-of-concept the first real-time 3D US-derived images of WSS using our recently-developed FV-IVUS matrix array^68^.

## Results

### CFD and US imaging in phantom geometries before stenting

**Fig. 1:**
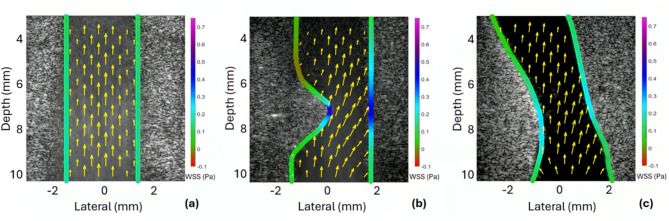
B-mode ultrasound images with vector Doppler and wall shear stress overlays of phantoms prior to stenting for **a** straight vessel phantom, **b** stenotic phantom with 55% stenosis, and **c** patient specific LAD vessel phantom. The mean WSS values for the middle segment are: **A** 0.0943 ± 0.0042 Pa (no stenosis), **B** 0.2750 ± 0.1489 Pa (55% stenosis), and **C** 0.1536 ± 0.0304 Pa (patient-specific stenosis).

The geometries used in both physical and in silico phantoms can be seen in the B-mode (grayscale) part of the US images prior to stenting (Fig. 1). While a uniformly low WSS is observed in the case of a straight geometry (Fig. 1a), a 55% stenosis (Fig. 1b) and a patient-specific stenosis (Fig. 1c) result in increased velocity and WSS near the stenosis. The 55% stenosis (Fig. 1b), which is more severe than the patient-specific stenosis in Fig. 1c, has the highest peak WSS (0.6423 Pa).

### CFD after stenting

**Fig. 2:**
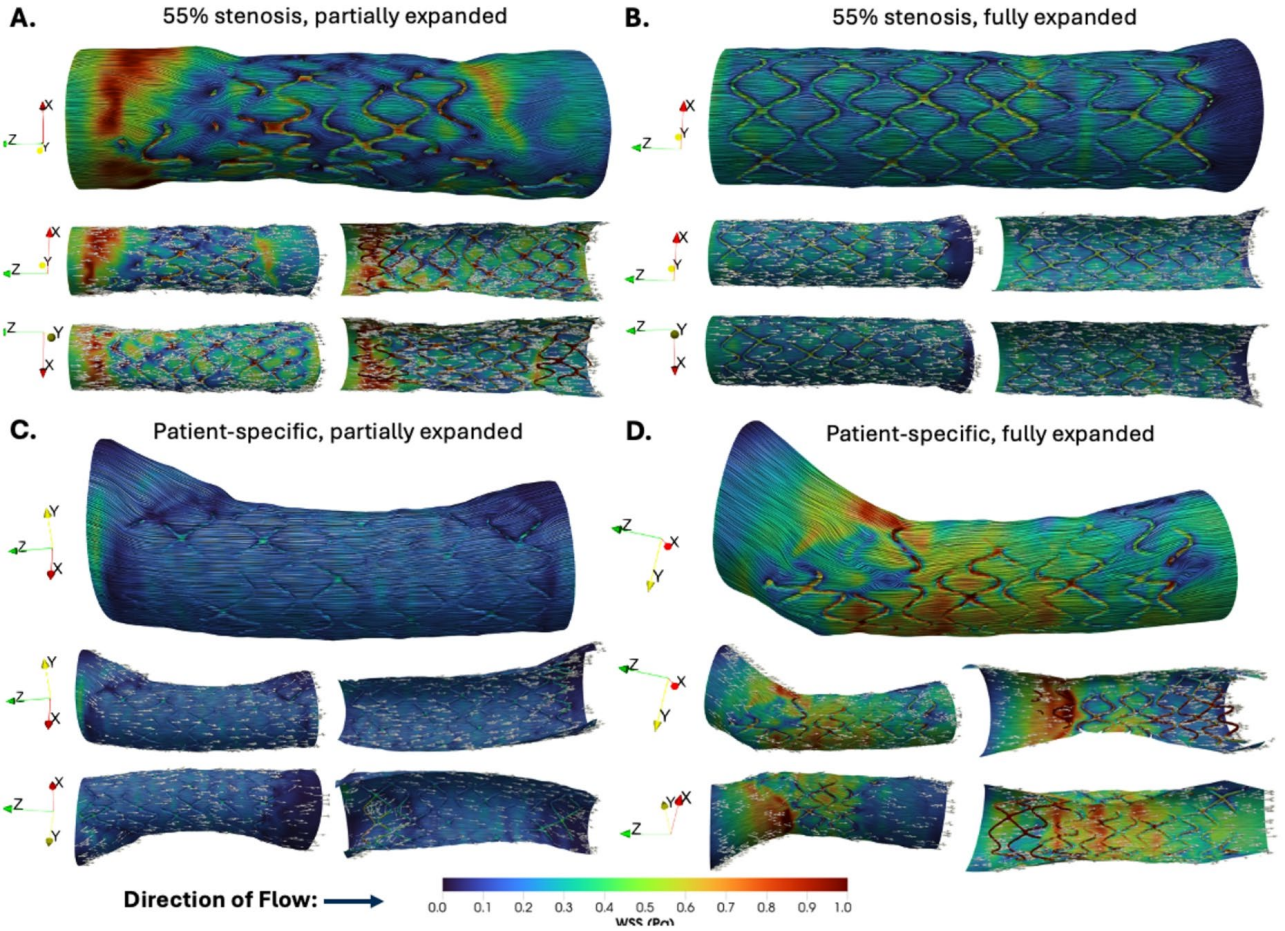
Wall shear stress (WSS) surface streamlines with normalized WSS vector glyphs for four stent deployment scenarios. The color corresponds to the magnitude of the WSS, and sparse white arrows in the lower panels indicate the direction of the WSS vector at each point on the surface for: **A** 55% stenosis, partially expanded stent; **B** 55% stenosis, fully expanded stent; **C** patient-specific geometry, partially expanded stent; and **D** patient-specific geometry, fully expanded stent. Multiple surface views and corresponding cross-sectional views are shown to highlight regions of WSS disturbance, flow separation, and reattachment.

The in silico velocity magnitude maps for the stented vessels are shown in Fig. 2 for a single cross-sectional plane located along the length of each vessel. For each case, the first image indicates the location of the cross-sectional plane in contact with the stent struts, and the second image shows a magnified version of the isolated velocity map. Maximum velocity values for Fig. 2 varied between 4.78 cm/s and 12.32 cm/s and were generally located near the center lumen of each vessel. The maximum velocity values were: (A) 12.32 cm/s for a 55% stenosis with a partially-expanded stent, (B) 5.28 cm/s for a 55% stenosis with a fully-expanded stent, (C) 9.19 cm/s for a patient-specific vessel geometry with a partially expanded stent, and (D) 4.78 cm/s for a patient-specific vessel geometry with a fully-expanded stent.

**Fig. 3:**
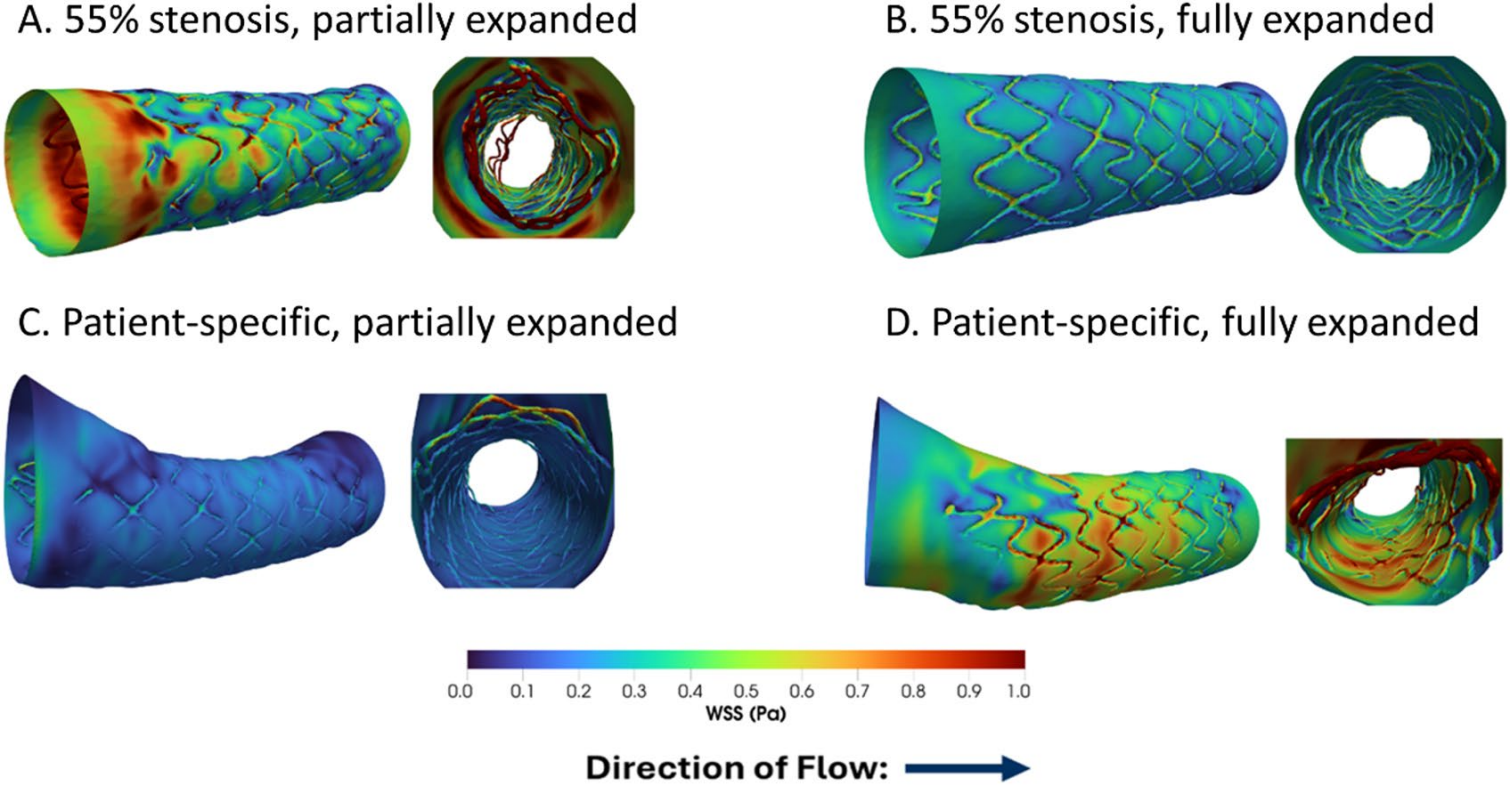
3D WSS contours in the region of interest (left) and cross-sectional views of the interior lumen (right) for **A** 55% stenosis with a partially expanded stent, **B** 55% stenosis with a fully-expanded stent, **C** Patient-specific vessel geometry with a partially expanded stent, and **D** Patient-specific vessel geometry with a fully expanded stent. The direction of flow is from left to right.

The in silico 3D WSS maps for the stented region of each vessel and a cross-sectional view of the interior lumen and stent struts are shown in Fig. 3. Peak WSS values range from 1.849 to 5.338 Pa, where the maximum values for each case were: (A) 5.338 Pa for a 55% stenosis with a partially expanded stent, (B) 3.209 Pa for a 55% stenosis with a fully-expanded stent, (C) 3.521 Pa for a patient-specific vessel geometry with a partially expanded stent, and (D) 1.849 Pa for a patient-specific vessel geometry with a fully-expanded stent. At locations of stent malapposition, the WSS on the inner surface was lower than in the surrounding lumen, often exhibiting a color pattern corresponding to the shape of the local partially expanded strut (Fig. 3C, proximal end). However, when large portions of the stent are severely malapposed towards the center of the vessel, locations of high WSS are also seen, likely due to local flow acceleration induced by the intertwining free-hanging struts (Fig. 3A and D).

### US imaging after stenting

**Fig. 4:**
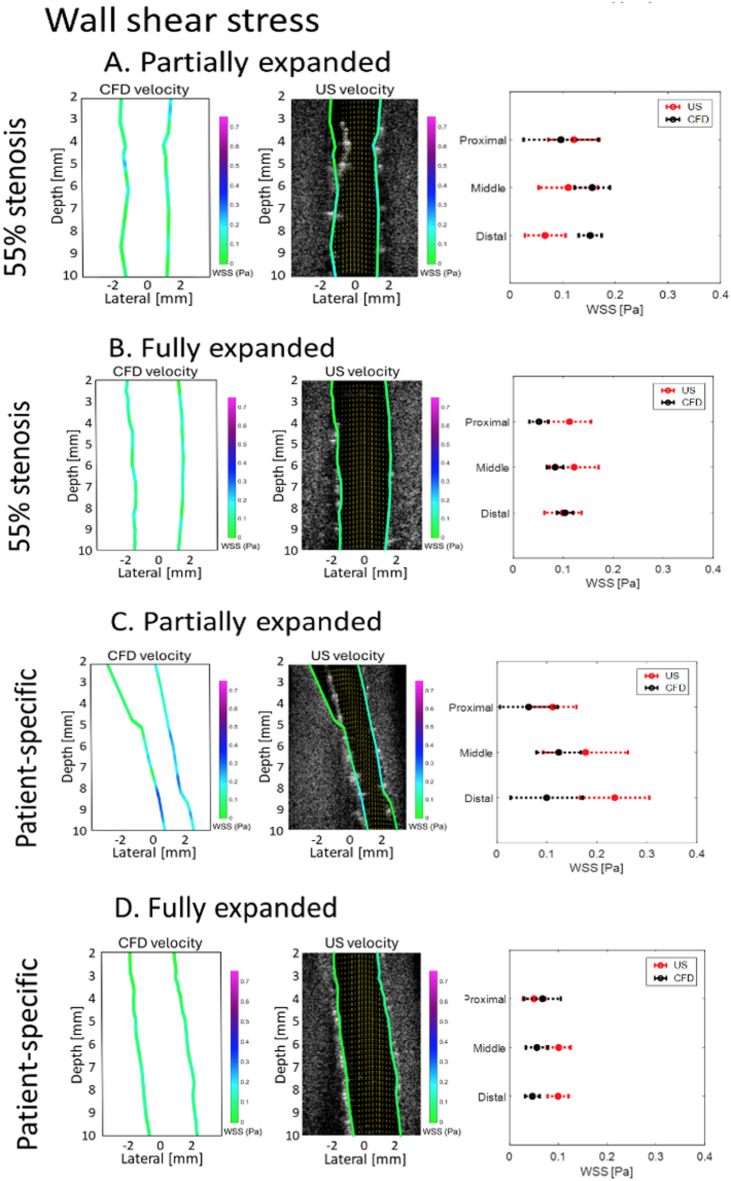
CFD and ultrasound-derived WSS maps for **A** a partially expanded stent in a 55% stenosis over 4 mm, and **B** the same geometry after the stent is fully expanded. In the bottom row, the ultrasound and CFD derived WSS maps are shown for **C** a partially expanded stent in a patient-specific geometry, and **D** the same geometry after the stent is fully expanded. The mean and standard deviation of values for each segment are shown on the right side. While the comparison here shows the difference in the WSS magnitude, the direction of the WSS vectors (tangent to the surface) estimated from US imaging was always consistent with the WSS from the CFD computations.

The velocity maps for all stented geometries are shown in Supplementary Fig. S2. The US-derived velocity maps are shown in the first column, followed by the corresponding 2D slice from the CFD-derived velocity maps in the second column for comparison. To the right of these two maps, the range of values is shown for both US and CFD for each segment (proximal, middle, and distal). Similarly, the WSS maps for all stented geometries are shown in Fig. 4.

#### Straight geometry

For a straight geometry with a fully expanded stent (not shown), the peak US and CFD velocities were 5.325 cm/s and 5.284 cm/s, respectively. Thus, US overestimated the peak velocity by 0.77% compared to CFD. When the velocity was analyzed by region, US underestimated the velocity compared to CFD by 11.83%, 0.25%, and 13.78% in the proximal, middle, and distal regions, respectively. The MAPE between US and CFD for this straight geometry case was 9.36%. The Spearman correlation coefficient between US-derived and CFD-derived velocities was 93.97%.

#### 55% stenosis

Considering velocity for a 55% stenosis with a partially expanded stent (see Supplementary Fig. S2 online), the peak US and CFD velocities were 10.119 cm/s and 9.601 cm/s, respectively. Thus, US overestimated the peak velocity by 5.12% compared to CFD. US underestimated the velocity by 6.41% in the proximal region. For the middle and distal regions, US overestimated the velocity by 6.71% and 5.75%, respectively. The MAPE between US and CFD for this case was 6.29%. The Spearman correlation coefficient was 92.47%.

Considering velocity for a 55% stenosis with a fully expanded stent, the peak US and CFD velocities were 6.787 cm/s and 7.207 cm/s, respectively (Supplementary Fig. S2). Thus, US underestimated the peak velocity by 5.83% compared to CFD. In the proximal and middle regions, US underestimated the velocity compared to CFD by 37.06% and 11.77%, respectively. In the distal region, US overestimated the velocity by 4.49%. The MAPE between US and CFD for this case was 17.77%. The Spearman correlation coefficient for US and CFD-derived velocity estimations was 83.13%.

Considering the WSS for a partially expanded stent (Fig. 4A), US underestimated WSS compared to CFD by 20.38% in the proximal region and overestimated WSS by 41.30% and 129.32% in the middle and distal regions, respectively. The MAPE between US-derived and CFD-derived WSS for this case was 63.63%. The spatial variances for US-derived and CFD-derived WSS were 0.0030 Pa^2^ and 0.0029 Pa^2^, respectively.

Considering the WSS for a fully expanded stent (Fig. 4B), US underestimated the WSS compared to CFD by 54.40% and 31.28% in the proximal and middle regions, respectively. For the distal region, US overestimated the mean WSS by 3.96%. The MAPE between US-derived and CFD-derived WSS for this case was 29.88%. The spatial variances for US-derived and CFD-derived WSS were 0.000764 Pa^2^ and 0.0020 Pa^2^, respectively.

#### Patient-specific geometry

Considering velocity for a patient-specific geometry with a partially-expanded stent (Supplementary Fig. S2), the peak US and CFD velocities were 7.749 cm/s and 9.186 cm/s, respectively. Thus, US underestimated the peak velocity by 15.64% compared to CFD. US overestimated the mean velocity compared to CFD by 18.13%, 12.07%, and 17.30% in the proximal, middle, and distal regions, respectively. Thus, the MAPE between US and CFD estimations for this case was 15.83%. The Spearman correlation coefficient was 82.43%.

Considering velocity for a patient-specific geometry with a fully expanded stent (Supplementary Fig. S2), the peak US and CFD velocities were 4.780 cm/s and 4.782 cm/s, respectively. Thus, US underestimated the peak velocity by 0.04% compared to CFD. US overestimated the velocity compared to CFD in the proximal and middle regions by 24.44% and 2.39%, respectively. In the distal third, US underestimated the mean velocity by 20.30%. Thus, the MAPE between US and CFD velocity estimations is 15.60%. The Spearman correlation coefficient for this case was 57.56%.

Considering WSS for a patient-specific geometry with a partially-expanded stent (Fig. 4C), US underestimated the WSS compared to CFD by 43.10%, 30.33%, and 57.80% in the proximal, middle, and distal regions, respectively. The MAPE between US-derived and CFD-derived WSS was 43.74%. The spatial variance for US-derived and CFD-derived WSS in this case were 0.0039 Pa^2^ and 0.0072 Pa^2^, respectively.

Considering WSS for a patient-specific geometry with a fully expanded stent (Fig. 4D), US overestimated the mean WSS compared to CFD by 34.8% in the proximal region and underestimated the mean WSS in both the middle and distal regions by 44.43% and 52.97%, respectively. The MAPE between US-derived and CFD-derived WSS was 44.07%. The spatial variance for US-derived and CFD-derived WSS in this case were 0.00079 Pa^2^ and 0.0011 Pa^2^, respectively.

Across all cases, the fully-expanded stent conditions show a consistent mean velocity through each region. Segments containing a partially expanded stent exhibited locally decreased mean WSS (0.0800 ± 0.0233 Pa vs. 0.1328 ± 0.0265 Pa, *p* = 0.0479) and increased mean spatial variance (0.0038 ± 0.0011 Pa^2^ vs. 0.00069 ± 0.000090 Pa^2^, *p* = 0.0546) compared to the fully expanded segments in the same acquisitions. The proximal region for all partially expanded cases showed a decrease in WSS at the wall associated with the malapposition compared to its middle and distal thirds. The proximal region for all partially expanded cases showed an increase in WSS at the wall opposite the malapposition. The straight geometry with a fully expanded stent (not shown) had the highest Spearman correlation of 93.97% between US and CFD velocity maps. For all velocity maps, the MAPE was 13.04 ± 4.82% with a mean Spearman correlation of 81.91 ± 14.59%.

#### 3D ultrasound-derived velocity and WSS estimation

**Fig. 5:**
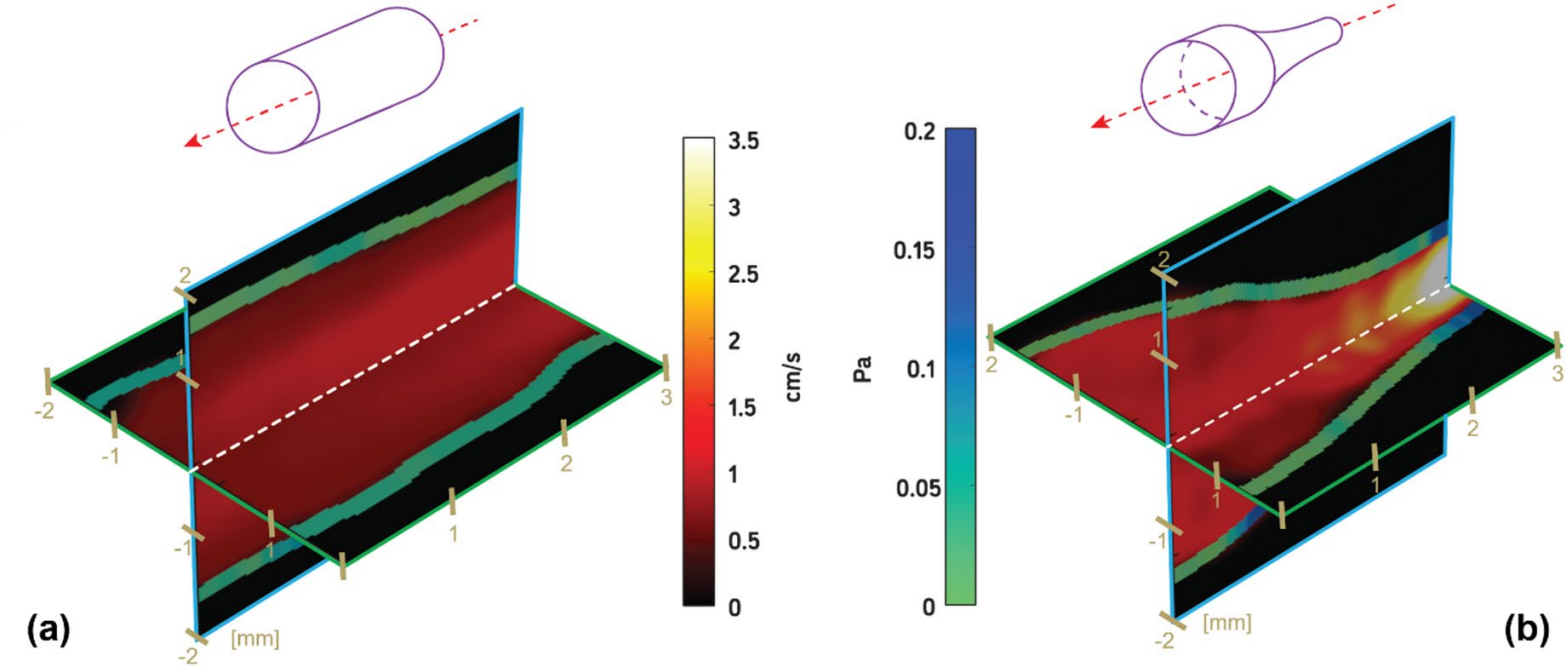
Real-time 3D ultrasound-derived velocity and WSS estimation in **a** straight geometry and **b** stenotic geometry. Peak velocity and WSS in (**a**) were 1.4 cm/s and 0.12 Pa, respectively. Peak velocity and WSS in (**b**) were 3.5 cm/s and 0.22 Pa, respectively.

The flow velocity and WSS are shown for two phantom geometries (straight and simple 50% stenosis) in Fig. 5. For the straight geometry in Fig. 5A, the peak velocity remained relatively constant through the middle of the artery and decreased towards the wall. For the stenosis in Fig. 5B, the peak velocity was located at and immediately downstream from the stenosis.

## Discussion

Accurate velocity profiles are important for estimating WSS; the US-derived velocity profiles and WSS estimates for the non-stented cases appear to be similar to WSS spatial distributions previously reported in literature^73,74^. The US-derived velocity maps for partially-expanded stents exhibited a decrease in WSS at the site of the malapposition, which is consistent with clinical studies investigating increased rate of restenosis at those locations^75–77^.

There are several potential sources of variation between US-derived and CFD-derived velocity and WSS maps. First, the CFD inlet boundary conditions are calibrated only on the flow rate and not on any spatially-dependent data and have an impact on the CFD solution that is only partially mitigated by the flow extensions. Thus, this improper boundary condition at the inlet would negatively impact downstream results^78^. This error could have a significant contribution to the variation observed between the US and CFD-derived data, particularly for the patient-specific case in which the inlet cross section is not circular. In addition, since imaging was only performed in a 2D plane, the US flow profile does not capture out-of-plane variation in flow velocities, which affects the comparison between CFD (based on 3D velocities) and 2D US reconstructions^78^.

Another potential source of error is the error in locating the wall of the physical phantom in the 2D US imaging data and also in the 3D segmentation of the micro-CT data. This is a well-known challenge, as small discrepancies in wall segmentation can significantly affect measurements of lumen diameter, flow velocity gradients, and therefore WSS. Furthermore, the stent generates metal artifacts in both US-based and micro-CT-based segmentations, however, this artifact is more severe in the reconstructed micro-CT volume.

Another consideration is that the geometrical complexity of the scenarios investigated here proves challenging to the numerical solvers. In this case, a consolidated commercial package (Ansys Fluent) was utilized, however, the accuracy of the solution is not fully guaranteed for these complex, stented geometries. In the future, more sophisticated mathematical tools may be needed to handle this complexity, for example, the Virtual Element Method (VEM)^79,80^ or a mesh-free approach such as physics-informed neural networks (PINNs)^81,82^. The extension of these methodologies to problems of this complexity is not trivial and will be pursued in follow-up studies.

In the present study, simulations are performed under the rigid-wall assumption and compared with phantoms having walls that are stiffer than in vivo arteries. In patient-specific computational hemodynamics, several factors—both physical and numerical—affect the overall accuracy of the results. On the physical side, geometric reconstruction and boundary conditions are considered more critical to the accuracy of results than fluid-structure interaction (FSI)^83,84^.

While incorporating artery stiffness via FSI would improve physical fidelity, it significantly increases the complexity of the simulations, particularly in the presence of a stent, which locally alters the mechanical properties of the vessel wall^85^. Conversely, Chiastra et al. compared the WSS computed for a rigid vs. compliant model in a simplified setting, concluding that the rigid assumption is adequate^86^. Therefore, given the geometric complexity in the presented cases, discrepancies between simulations and measurements are more likely attributable to geometric and numerical factors rather than to the rigid-wall assumption alone.

Finally, registration of the 2D US plane to the 3D CFD domain may introduce variability if there is registration error. The magnitude of the geometric mismatch is approximately 0.25 to 0.5 mm. Given the magnitude of this error, it is estimated that the produced variation in the WSS is approximately 4.17 ± 3.66%. Specifically, slight angular rotations in the plane near the transducer during registration will produce increasingly larger errors moving towards the distal end of the plane. In the partially expanded cases, the struts provide anatomical landmarks that aid registration, while the fully expanded cases have greater radial symmetry and lack unique features, which increases the difficulty of accurate registration.

In the future, this can be addressed by using a two-dimensional matrix array to acquire the flow map parallel to the array at the inlet, then applying this map to the inlet boundary conditions for the CFD simulation. For segmentation errors, methods for increasing contrast between the lumen and phantom to reduce metal artifacts are currently being explored to automate the 3D segmentation of the phantom and improve the accuracy of wall identification. Finally, additional fiducial markers placed in the phantom during phantom development could aid in registration.

The non-stented straight vessel had a nearly constant flow profile with respect to depth (Fig. 1A), as expected with steady flow. Thus, the WSS values were also approximately constant with respect to depth. The fully expanded straight case (not shown) had the highest correlation between velocity trends (93.97%). This was expected, as this was the simplest stented case tested. The discrepancies observed in the proximal and distal regions were most likely due to a combination of the sources of error previously discussed. The decrease in velocity near the distal end may be due to decreased SNR farther away from the transducer. The decrease in the US-derived velocity map at the proximal end for the fully expanded case may be due to the influence of the transducer restricting flow at the outlet. Despite these discrepancies, the velocity maps agree quantitatively, with both velocity and WSS map exhibiting similar trends.

For the 55% stenosis with a fully expanded stent (Supplementary Fig. S2, Fig. 4B), trends between velocity and WSS values are quite similar, with the exception of the proximal region for the velocity map. Again, this may be caused by the proximity between the face of the transducer and the vessel outlet. The 55% stenosis with a partially expanded stent (Supplementary Fig. S2, Fig. 4A) also had strong agreement in velocity (92.47%) and WSS trends. However, the CFD WSS contains locally-elevated WSS at certain locations. One possible reason is that the US WSS estimation technique lacked sufficient resolution or sensitivity to detect these elevated points around stent struts, leading to an underestimation of peak values. Alternatively, the peaks may have been smoothed during the US-based WSS estimation process, causing a reduction in the observed maximum WSS.

The patient-specific geometry exhibited similar trends prior to stenting (Fig. 1C) to that of the non-stented 55% stenosis geometry (Fig. 1B). For the partially expanded and fully expanded stent cases with the patient-specific geometry, the WSS trends agree between US and CFD (Fig. 4C-D). The velocity trends and values were also similar for the patient-specific partially expanded case (82.43%). The patient-specific geometry with a fully expanded stent had the lowest agreement, with a correlation coefficient of 57.56%. The spatial shift in the peak velocities in part D of Supplementary Fig. S2 could be the result of the limited field of view in micro-CT images. The 13 mm FOV may not have captured enough of the vessel tortuosity, since the patient-specific geometry contained over 20 mm of reconstructed CT-angiography data.

The trends in the 3D US-derived velocity map (Fig. 5) were as expected, with the highest velocities at the lowest MLA and immediately proximal to the MLA. Congruently, the peak WSS in Fig. 5 was located at the lowest MLA.

There are a few limitations to this work. First, continuous (non-pulsatile) flow was used in the experiments, as locally low TA-WSS has been demonstrated as an indicator that is co-localized with plaque formation and rate of progression^75–77^. Regardless, coronary flow is physiologically pulsatile, and the steady-flow experimental configuration represents a simplification that does not capture transient WSS dynamics present in vivo. Similarly, the physiological motion of the heart was not modeled in the in vitro flow phantom platform. These displacements in the vessel from cardiac motion can contribute to erroneous WSS estimations^87^. Thus, future work will investigate WSS estimation in stented coronary geometries with pulsatile flow and cardiac motion, which we have previously investigated without stenting^88^. While we have previously demonstrated cardiac motion compensation in order to estimate WSS in 2D^88^, motion compensation strategies for blood flow imaging^89–91^ have not been implemented in 3D.

Another limitation was the use of degassed water with microbubbles instead of blood or a blood-mimicking solution, which does not fully replicate the non-Newtonian rheological behavior of blood or the interaction between red blood cells and the vessel wall. Finally, 2D US was used to estimate the WSS but fails to capture the entire coronary environment. Utilizing our forward-viewing matrix array allows for 3D WSS estimation (Fig. 5), which is preferable because all velocity components would be used to estimate WSS, yielding a more accurate estimation^27^. This would also allow 3D volumetric flow measurements, which may represent an additional useful marker in this application^92–94^. Overall, these limitations are inherent to the in vitro flow phantom platform used in this study, which cannot fully replicate in vivo pulsatile flow, cardiac motion, or blood rheology.

To our knowledge, this is the first demonstration of using US to accurately map WSS in stented coronary artery geometries, with CFD providing validation of the accuracy of US-derived WSS. The agreement in trends between US-derived and CFD-derived velocity and WSS maps further supports the feasibility of FV-IVUS to guide PCI with the appropriate FV-IVUS array. We also presented the first real-time 3D US-derived WSS estimation in coronary geometries in phantoms by applying the described methods to the acquired 2D slices of the 3D data set (Fig. 5). Accurately estimating WSS during PCI would allow for real-time stent optimization based on local hemodynamics. Stents are currently optimized with intracoronary anatomical imaging and wire-based physiological measurements to improve patient outcomes^10,19^. However, FV-IVUS could provide real-time 3D imaging of anatomy and physiology to optimize stent placement, which could reduce rates of restenosis and MACE. Furthermore, FV-IVUS could improve risk stratification post-PCI to aid in refining treatment plans^31^. For example, patients with significantly lower WSS around the stent struts may be considered for earlier follow-up compared to standard practice.

In addition, in the future, we plan to use data assimilation techniques^95,96^ to combine US data and CFD simulations. Such an approach would utilize mathematically sound techniques to rigorously convert the available (but potentially limited) US data in the imaging field of view to accurate boundary information to be used in the patient-specific model.

## Methods

### Development of coronary artery phantoms

To evaluate the feasibility of US-based wall shear stress (WSS) estimation in stented vessels, four sets of experiments were conducted with vessel-mimicking phantoms having different geometries. A total of 11 physical phantoms were created across all 4 experiments. The first three experiments each included three phantoms of identical geometry but varying stent conditions: no stent, partially expanded stent, and fully expanded stent. US imaging-derived velocity and WSS estimates were then compared with CFD. The first phantom contained a straight 3.2 mm-diameter vessel geometry, simulating an adult proximal left anterior descending (LAD) coronary artery^97^. The second phantom geometry contained a stenotic vessel geometry with an eccentric stenosis of 55% in diameter over a distance of 4 mm. A reduction in diameter > 50% corresponds to a 75% reduction in the cross-sectional area, which is an established threshold for significant ischemia^98–100^. However, only ~ 30% of stenoses greater than 50% cause clinically significant ischemia, complicating treatment decisions^101,102^. The third phantom geometry contained a de-identified patient-specific geometry reconstructed from CT angiography of the LAD coronary artery. This isolated stenotic segment from de-identified patient-specific CTA had a slight curvature. For these three geometries (straight, 55% stenosis, and patient-specific), physical phantoms were developed (described below), and then 2D ultrasound images were acquired for comparison with CFD in the same geometries.

Finally, the goal of the fourth experiment was to demonstrate initial proof-of-concept with the newly-developed FV-IVUS array transducer for WSS imaging. This experimental setup was only used for 3D US imaging and utilized two physical phantoms: a straight 3 mm-diameter geometry and a 3 mm-diameter geometry with a 50% diameter concentric stenosis. Each phantom was imaged with the FV-IVUS matrix array, and 3D WSS maps were generated by estimating 2D WSS maps on a slice-by-slice basis.

All geometries were 3D printed with a resolution of 25 μm using a FormLabs Form 3 + resin printer. Silicone rubber molds (Mold Star 15 Slow, Smooth-On, Inc., Macungie, PA) were cast around the prints, then filled with carnauba wax (PremiumCraft, Minneapolis, MN), a water-soluble wax. Once solidified, a gelatin-based tissue-mimicking material was poured around the wax to form the phantom. The tissue-mimicking material consisted of 7.5% gelatin, 6% graphite for acoustic scattering (3–6 μm, Graphite Powder, Besucce, China), 5% n-propanol, and 81.5% water by volume^103^. An additional 3 ml of glutaraldehyde were added to 7 ml of water and then added to the phantom mixture to mimic vessel-wall acoustic properties^104^. After congelation, the wax was dissolved and the luminal geometry was confirmed by acquiring micro-computed tomography (micro-CT) scans (µ-CT 50, Scanco Medical AG., Zürich, Switzerland) for comparison with the original CT-angiography data.

For each geometry (straight, stenotic, and patient-specific) in the first three experiments, three phantoms were created: native, partially expanded stent, and fully expanded stent. In stented cases, a stent and balloon catheter (Medtronic Resolute Integrity RX Zotarolimus-Eluting Coronary Stent System, Medtronic, Minneapolis, MN) were positioned across the stenosis and deployed following standard techniques^18,105^. For the partially expanded cases, US imaging was used to confirm a malapposition of approximately 25% (0.8 mm). This ensured a malapposition greater than the 0.4 mm threshold, which requires stent adjustment or repeat revascularization due to increased risk for MACE^106^.

### Ultrasound measurements

**Fig. 6:**
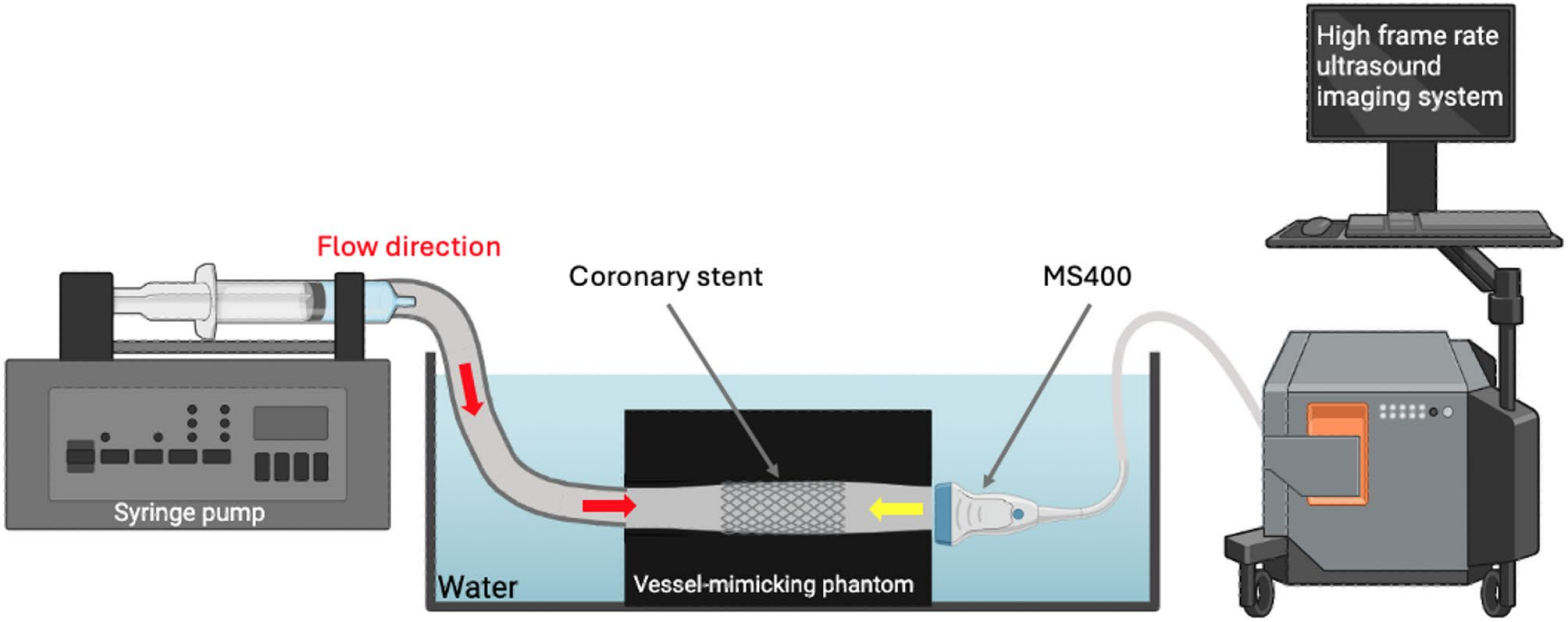
Diagram of the experimental setup. Vessel-mimicking phantom with a stent submerged in degassed-water. Microbubble solution is infused at 20 ml/min with a syringe pump. A commercial high-frequency linear array (MS400, VisualSonics) is positioned 1 mm from the outlet. The yellow arrow indicates US beam direction, and the red arrows indicate the flow direction. Created with BioRender.com.

All phantoms in the first three experiments were submerged in degassed water for imaging with a high frequency linear array (MS400, FUJIFILM VisualSonics, Inc. Toronto, Canada) positioned 1 mm in front of the vessel outlet and aligned coaxially with the lumen (Fig. 6). The transducer was connected to a research US system (Verasonics Vantage 256, Kirkland, Washington, USA). A microbubble solution (2.5 × 10^6^ microbubbles per ml) was continuously infused using a syringe pump (PHD 2000, Harvard Apparatus, Holliston, Massachusetts), approximating an in vivo bolus injection of Definity^®^ microbubbles in a 75-kg person, assuming 3 L of plasma within 5 L of whole blood^107^. A continuous flow rate of 20 mL/min was used, approximating average LAD flow^108,109^. Continuous flow was utilized because time-averaged wall shear stress (TAWSS) has been shown to co-locate with locally low WSS, with both promoting plaque formation and rate of progression^75–77,110–112^. Computational studies confirm that TAWSS is consistent with WSS over one heartbeat^96^.

US imaging was performed with three unfocused plane steered plane wave events (−8°, 0°, and + 8°) transmitted at 23.44 MHz with 2-cycle pulses. The post-compounding frame rate was 10,000 fps. 1100 frames of in-phase and quadrature (IQ) data were recorded for each acquisition, and acquisitions were performed in duplicate.

**Fig. 7:**
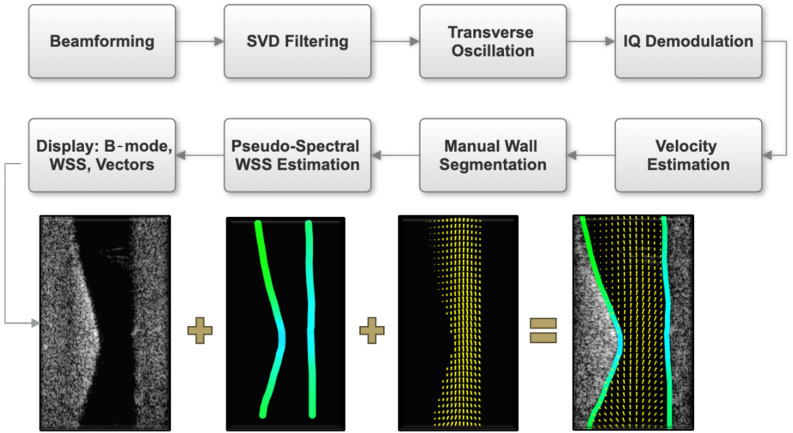
Post-processing workflow from US acquisition to velocity and wall shear stress estimation.

The tissue signal was first removed by applying singular value decomposition (SVD) filtering to the beamformed RF data (Fig. 7)^113^. If we denote by $\:M$ the matrix of the images, we apply SVD to decompose $\:M$ as:

1 $$\:M=U\:\varSigma\:\:{V}^{T}$$

where $\:U$ and $\:V$ are orthogonal and $\:\:\varSigma\:$ is diagonal. The entries of $\:\varSigma\:$ are the singular values. The largest singular value was discarded to remove stationary echoes. Transverse oscillation was introduced in the beamformed RF data for lateral velocity estimation^114–116^. The filtered data was then IQ demodulated and used to estimate the flow velocity through the lumen in both axial and lateral directions. The lateral velocities were estimated with a fourth-order autocorrelation estimator, and the axial velocities were estimated with a first-order autocorrelation estimator^114–116^. An ensemble size of 1100 frames was used. Velocities were smoothed with MATLAB’s (MATLAB, Version 23.2, R2023b) “smooth” function using the loess method and 10% data span. Loess applies a weighted local regression with tri-cubic weights and minimizes weighted least squares error within the span, which aids in noise reduction and local adaptivity.

### Wall shear stress estimation

Smoothed axial and lateral velocities were used to estimate the WSS with a pseudo-spectral approach previously developed by our group^27,88^. The microbubble solution was assumed to be Newtonian and non-compressible, and thus constant in viscosity. The steady Navier-Stokes equations for an incompressible fluid arises from the principles of momentum and mass conservation (e.g^84^., :

2 $$\:\left\{\begin{array}{c}\rho\:\left(\boldsymbol{u}\cdot\:\nabla\:\right)u+\nabla\:\cdot\:\sigma\:+\nabla\:p=0\\\:\nabla\:\cdot\:\:u=0\end{array}\right.$$

where $\:\boldsymbol{u}$ is the velocity, $\:p$ is the pressure, $\:\rho\:$ is the density, $\:\nabla\:\cdot\:$ denotes the divergence operator, and the deviatoric stress tensor $\:\boldsymbol{\sigma\:}$ is:

3 $$\:\boldsymbol{\sigma\:}=-\mu\:(\nabla\:\boldsymbol{u}+\nabla\:{\boldsymbol{u}}^{T})$$

where *µ* is the viscosity. The steady version of the Navier-Stokes equations was implemented since this is the case considered in the experiments. The tangential component of the deviatoric stress tensor is the WSS defined as:

4 $$\:WSS=\boldsymbol{\sigma\:}\cdot\:\boldsymbol{n}-\left(\boldsymbol{n}\cdot\:\boldsymbol{\sigma\:}\cdot\:\boldsymbol{n}\right)\boldsymbol{n}$$

where $\:\boldsymbol{n}$ is the normal unit vector outward to the boundary. The boundary was identified as the first zero-velocity value outside of the velocity data. A 2nd degree polynomial was fit locally to the edge pixels with weighted least squares regression. This is a more robust approach to minimize the effects of clutter and noise at the wall. A Fourier sine series interpolation was used at each depth for each lateral and axial velocity profiles. The lateral velocities were smoothed with a 3% moving average prior to differentiating the function due to higher sensitivity to noise. Lateral velocity components were included because this improves WSS estimation accuracy, and a Fourier approximation is used because it is more robust to noise than polynomial fitting^27^.

### Segmentation, meshing, and CFD set-up

Each phantom from the first three experiments was scanned with a micro-CT scanner after staining for 24 h with a 30% solution of Optiray 320 (Guerbet LLC, Princeton, NJ) to enhance lumen-phantom contrast and reduce strut artifacts. Acquisition parameters were 90 kVp, 145 µA, 750 µs integration, and 3 averaging events. Each scan yielded 271 slices at a voxel size of 49 μm.

After reconstruction, phantoms were semi-automatically segmented with Dragonfly 3D World (Comet Technologies, Wünnewil-Flamatt, Switzerland). Multi-ROI labeling differentiated between the lumen, stent, and phantom wall. The stent struts were automatically segmented with intensity thresholding (lower bound: µ ≈ 12738 cm⁻¹; upper: max intensity). The lumen-phantom interface was semi-automatically segmented with a local-Otsu filter and partial manual segmentation. The local-Otsu filter binarizes the pixels within the scope of the selection operator (0.200 mm Ø – 2 mm Ø) to either foreground or background by dynamically thresholding the local intensity histogram. Manual segmentation was used where metal artifacts interfered with the local-Otsu filter.

Finally, the stent was subtracted from the stented lumen using a Boolean operation in Meshlab^117^, and resulting degenerate/self-intersecting triangles were repaired with MeshLab^117^ and Autodesk NetFabb^118^. The result was re-meshed isotropically with MeshLab to obtain a surface reticulation amenable to volumetric meshing. Flow extensions, 3× the reference diameter, were added with VMTK (Vascular Modeling ToolKit)^119^ to morph the inlet to a circular profile, due to the lack of precise inflow velocity data. This allowed application of a Poiseuille profile at the inlet, alleviating the impact of partially arbitrary boundary conditions while maintaining the experimental flow rate of 20 ml/min. At the outflow, the traction-free condition was prescribed, i.e. $\:p\boldsymbol{n}\:+\sigma\:\cdot\:\:\boldsymbol{n}\:=\:0$, as this has minimal impact on the numerical solution^120^. A final repair and re-meshing were performed before volumetric meshing with first-order tetrahedra in ICEM CFD (ANSYS ICEM 21, Ansys Inc, Canonsburg, PA, USA), tuning element size to resolve stent struts, yielding 1.0–1.4.0.4 million elements. CFD simulations were performed with a finite volume-based discretization implemented in the Ansys Fluent solver (ANSYS 21, Ansys Inc, Canonsburg, PA, USA), using a pressure-based coupled algorithm with a second-order upwind scheme for the spatial discretization, with residual criteria set to $\:{10}^{-5}$. Each simulation required approximately 10–20 min to complete on a high-end workstation (12 core, 24 processor Intel i9-9920X @ 3.50 GHz, 128 GB Total Memory). Visualization of all CFD results were done with *ParaView* (Kitware Inc., Clifton Park, NY, USA).

### Registration and comparison between ultrasound and CFD maps

To register the 3D CFD data with the 2D ultrasound data, the coherent point drift (CPD) and iterative closest point (ICP) algorithms were used in succession in the open-source software CloudCompare (Telecom ParisTech & Électricité de France, France). Point clouds were downsampled by 30% to improve computational efficiency. CFD data was considered the stationary source, and US data was considered the moving source. The US data was translated and rotated to minimize the distance between points within the point clouds until either 600 iterations were achieved or the convergence criteria of 10^− 5^ was reached. Both CPD and ICP were chosen for registration because they are robust against noise^121,122^. Post registration, the 3D CFD-derived WSS and velocity fields were projected onto the aligned 2D plane using nearest-neighbor interpolation in CloudCompare. The planes were then exported and compared in MATLAB 2024b (Natick, Massachusetts, United States).

2D US-derived velocity and WSS maps were compared with CFD in all cases. The mean absolute percentage error (MAPE), and Spearman correlation were calculated for each region in each velocity map.

## Supplementary Information

Below is the link to the electronic supplementary material.

Supplementary Material 1

## Data Availability

The data acquired is available upon reasonable request to the corresponding author.

## References

[1] Khan, M. A. et al. Global epidemiology of ischemic heart disease: Results from the Global Burden of Disease Study. *Cureus* **12**(7), e9349 (2020). 32742886 10.7759/cureus.9349 PMC7384703

[2] Ralapanawa, U. & Sivakanesan, R. Epidemiology and the magnitude of coronary artery disease and acute coronary syndrome: A narrative review. *J. Epidemiol. Glob. Health* **11**(2), 169–177 (2021). 33605111 10.2991/jegh.k.201217.001 PMC8242111

[3] Bentzon, J. F. et al. Mechanisms of plaque formation and rupture. *Circ. Res.* **114** (12), 1852–1866 (2014). 24902970 10.1161/CIRCRESAHA.114.302721

[4] Shahjehan, R. D., Sharma, S. & Bhutta, B. S. Coronary Artery Disease. In *StatPearls* (Treasure Island, FL, 2025). 33231974

[5] Gerber, Y. et al. Coronary revascularization in the community. A population-based study, 1990 to 2004. *J. Am. Coll. Cardiol.* **50** (13), 1223–1229 (2007). 17888838 10.1016/j.jacc.2007.06.022

[6] Jennings, S. et al. Trends in percutaneous coronary intervention and angiography in Ireland, 2004–2011: Implications for Ireland and Europe. *Int. J. Cardiol. Heart Vessel*. **4**, 35–39 (2014). 29450183 10.1016/j.ijchv.2014.08.001 PMC5802397

[7] Head, S. J. et al. Current practice of state-of-the-art surgical coronary revascularization. *Circulation* **136**(14), 1331–1345 (2017). 28972063 10.1161/CIRCULATIONAHA.116.022572

[8] Liu, H. et al. A systematic review and meta-analysis of 35,409 patients undergoing PCI versus CABG for unprotected left main coronary artery diseases. *Rev. Cardiovasc. Med.* **25**(8), 282 (2024). 39228473 10.31083/j.rcm2508282 PMC11367015

[9] Bajeu, I. T. et al. Intrastent restenosis: A comprehensive review.. *Int. J. Mol. Sci.*10.3390/ijms25031715 (2024). 38338993 10.3390/ijms25031715 PMC10855438

[10] Choi, K. H. et al. Prognostic Impact of Operator Experience and IVUS Guidance on Long-Term Clinical Outcomes After Complex PCI. *JACC Cardiovasc. Interv*. **16** (14), 1746–1758 (2023). 37495350 10.1016/j.jcin.2023.04.022

[11] Dangas, G. D. et al. In-stent restenosis in the drug-eluting stent era.. *J. Am. Coll. Cardiol.* **56**(23), 1897–907 (2010). 21109112 10.1016/j.jacc.2010.07.028

[12] Groenland, F. T. W. et al. Intravascular ultrasound-guided versus coronary angiography-guided percutaneous coronary intervention in patients with acute myocardial infarction: A systematic review and meta-analysis.. *Int. J. Cardiol.* **353**, 35–42 (2022). 35041893 10.1016/j.ijcard.2022.01.021

[13] Mauri, L. et al. Long-term clinical outcomes after drug-eluting and bare-metal stenting in Massachusetts. *Circulation* **118** (18), 1817–1827 (2008). 18852368 10.1161/CIRCULATIONAHA.108.781377 PMC2821087

[14] Zahn, R. et al. Incidence and predictors of target vessel revascularization and clinical event rates of the sirolimus-eluting coronary stent (results from the prospective multicenter German Cypher Stent Registry). *Am. J. Cardiol.* **95**(11), 1302–8 (2005). 15904633 10.1016/j.amjcard.2005.01.072

[15] Xu, N. et al. Five-year outcomes of biodegradable versus second-generation durable polymer drug-eluting stents used in complex percutaneous coronary intervention. *Chin. Med. J. (Engl.)* **136**(3), 322–330 (2023). 36848178 10.1097/CM9.0000000000002450 PMC10106121

[16] Ding, D. et al. Immediate post-procedural functional assessment of percutaneous coronary intervention: Current evidence and future directions. *Eur. Heart J.* **42**(27), 2695–2707 (2021). 33822922 10.1093/eurheartj/ehab186

[17] Byrne, R. A. et al. 2023 ESC Guidelines for the management of acute coronary syndromes. *Eur. Heart J.* **12** (38), 3720–3826 (2023). 10.1093/eurheartj/ehad191 37622654

[18] Lawton, J. S. et al. 2021 ACC/AHA/SCAI guideline for coronary artery revascularization: A report of the American College of Cardiology/American Heart Association Joint Committee on clinical practice guidelines. *Circulation* **145**(3), e18–e114 (2022). 34882435 10.1161/CIR.0000000000001038

[19] Park, H. et al. Optimal stenting technique for complex coronary lesions: intracoronary imaging-guided pre-dilation, stent sizing, and post-dilation. *JACC Cardiovasc. Interv* **13**(12), 1403–1413 (2020). 32473888 10.1016/j.jcin.2020.03.023

[20] Jones, D. A. et al. Angiography alone versus angiography plus optical coherence tomography to guide percutaneous coronary intervention: outcomes from the Pan-London PCI cohort. *JACC Cardiovasc. Interv* **11**(14), 1313–1321 (2018). 30025725 10.1016/j.jcin.2018.01.274

[21] Mintz, G. S. Clinical utility of intravascular imaging and physiology in coronary artery disease. *J. Am. Coll. Cardiol.* **64** (2), 207–222 (2014). 24530669 10.1016/j.jacc.2014.01.015

[22] Maehara, A. et al. IVUS-guided versus OCT-guided coronary stent implantation: A critical appraisal. *JACC Cardiovasc. Imaging* **10**(12), 1487–1503 (2017). 29216976 10.1016/j.jcmg.2017.09.008

[23] De Bruyne, B. et al. Fractional flow reserve-guided PCI for stable coronary artery disease. *N Engl. J. Med.* **371** (13), 1208–1217 (2014). 25176289 10.1056/NEJMoa1408758

[24] van Zandvoort, L. J. C. et al. Improving PCI outcomes using postprocedural physiology and intravascular imaging. *JACC Cardiovasc. Interv* **14**(22), 2415–2430 (2021). 34794649 10.1016/j.jcin.2021.08.069

[25] Ng, J. et al. Local hemodynamic forces after stenting implications on restenosis and thrombosis. *Arterioscler. Thromb. Vascular Biology* **37**(12), 2231–2242 (2017). 10.1161/ATVBAHA.117.309728 29122816

[26] Truesdell, A. G. et al. Intravascular imaging during percutaneous coronary intervention: JACC state-of-the-art review. *J. Am. Coll. Cardiol.* **81**(6), 590–605 (2023). 36754518 10.1016/j.jacc.2022.11.045

[27] Tempestti, M. A pseudo-spectral method for wall shear stress estimation from Doppler ultrasound imaging in coronary arteries. *Cardiovasc. Eng. Technol.* **15**(6), 647–666 (2024). 39103664 10.1007/s13239-024-00741-2 PMC13338701

[28] Katritsis, D. et al. Wall shear stress: theoretical considerations and methods of measurement. *Prog Cardiovasc. Dis.* **49** (5), 307–329 (2007). 17329179 10.1016/j.pcad.2006.11.001

[29] Candreva, A. et al. Impact of endothelial shear stress on coronary atherosclerotic plaque progression and composition: A meta-analysis and systematic review. *Int. J. Cardiol.* **407**, 132061 (2024). 38641263 10.1016/j.ijcard.2024.132061

[30] Papafaklis, M. I. et al. Effect of the local hemodynamic environment on the de novo development and progression of eccentric coronary atherosclerosis in humans: insights from PREDICTION. *Atherosclerosis* **240** (1), 205–211 (2015). 25801012 10.1016/j.atherosclerosis.2015.03.017

[31] Stone, P. H. et al. Role of low endothelial shear stress and plaque characteristics in the prediction of nonculprit major adverse cardiac events: The PROSPECT study. *JACC Cardiovasc. Imaging* **11**(3), 462–471 (2018). 28917684 10.1016/j.jcmg.2017.01.031

[32] Chiastra, C. et al. Coronary artery stenting affects wall shear stress topological skeleton.. *J. Biomech. Eng.*10.1115/1.4053503 (2022). 35015058 10.1115/1.4053503

[33] Candreva, A. et al. Influence of intracoronary hemodynamic forces on atherosclerotic plaque phenotypes. *Int. J. Cardiol.* **399**, 131668 (2024). 38141723 10.1016/j.ijcard.2023.131668

[34] Cecchi, E. et al. Role of hemodynamic shear stress in cardiovascular disease. *Atherosclerosis* **214** (2), 249–256 (2011). 20970139 10.1016/j.atherosclerosis.2010.09.008

[35] Dolan, J. M., Kolega, J. & Meng, H. High wall shear stress and spatial gradients in vascular pathology: a review. *Ann. Biomed. Eng.* **41** (7), 1411–1427 (2013). 23229281 10.1007/s10439-012-0695-0 PMC3638073

[36] Eshtehardi, P. et al. High wall shear stress and high-risk plaque: an emerging concept. *Int. J. Cardiovasc. Imaging*. **33** (7), 1089–1099 (2017). 28074425 10.1007/s10554-016-1055-1 PMC5496586

[37] Salmasi, M. Y. et al. High wall shear stress can predict wall degradation in ascending aortic aneurysms: An integrated biomechanics study. *Front. Bioeng. Biotechnol.* **9**, 750656 (2021). 34733832 10.3389/fbioe.2021.750656 PMC8558434

[38] Elliott, M. C., Blair, J. & Menary, R. Highlighting hemodynamic risks for bioresorbable stents in coronary arteries. *Fluids* **8**(9), 241 (2023).

[39] Kumar, S. et al. Stent underexpansion is associated with high wall shear stress: a biomechanical analysis of the shear stent study. *Int. J. Cardiovasc. Imaging*. **39** (7), 1375–1382 (2023). 37119348 10.1007/s10554-023-02838-6

[40] Shah, I. et al. Impact of the stent footprint on endothelial wall shear stress in patient-specific coronary arteries: A computational analysis from the SHEAR-STENT trial. *Comput. Methods Programs Biomed.* **266**, 108762 (2025). 40245606 10.1016/j.cmpb.2025.108762

[41] Wu, W. et al. Hemodynamic microenvironment of coronary stent strut malapposition. *Comput. Biol. Med.* **184**, 109378 (2025). 39549532 10.1016/j.compbiomed.2024.109378

[42] Carrizo, S. et al. Functional assessment of coronary artery disease by intravascular ultrasound and computational fluid dynamics simulation. *Rev. Port Cardiol.* **33** (10), 645e1–645e4 (2014). 10.1016/j.repc.2014.03.013 25441999

[43] Sun, Z. & Xu, L. Computational fluid dynamics in coronary artery disease. *Comput. Med. Imaging Graph.* **38**(8), 651–663 (2014). 25262321 10.1016/j.compmedimag.2014.09.002

[44] Zhang, J. M. et al. Perspective on CFD studies of coronary artery disease lesions and hemodynamics: a review. *Int. J. Numer. Method Biomed. Eng.* **30** (6), 659–680 (2014). 24459034 10.1002/cnm.2625

[45] Candreva, A. et al. Current and future applications of computational fluid dynamics in coronary artery disease. *Rev. Cardiovasc. Med.* **23**(11), 377 (2022). 39076179 10.31083/j.rcm2311377 PMC11269074

[46] Morris, P. D. et al. Virtual fractional flow reserve from coronary angiography: Modeling the significance of coronary lesions: Results from the VIRTU-1 (VIRTUal Fractional Flow Reserve From Coronary Angiography) study.. *JACC Cardiovasc. Interv.* **6**(2), 149–57 (2013). 23428006 10.1016/j.jcin.2012.08.024

[47] Balossino, R. et al. Effects of different stent designs on local hemodynamics in stented arteries. *J. Biomech.* **41** (5), 1053–1061 (2008). 18215394 10.1016/j.jbiomech.2007.12.005

[48] Beier, S. et al. Hemodynamics in idealized stented coronary arteries: Important stent design considerations. *Ann. Biomed. Eng.* **44**(2), 315–329 (2016). 26178872 10.1007/s10439-015-1387-3 PMC4764643

[49] Duraiswamy, N., Schoephoerster, R. T. & Moore, J. E. Jr. Comparison of near-wall hemodynamic parameters in stented artery models. *J. Biomech. Eng.* **131** (6), 061006 (2009). 19449960 10.1115/1.3118764 PMC2767376

[50] LaDisa, J. F. Jr. et al. Stent design properties and deployment ratio influence indexes of wall shear stress: a three-dimensional computational fluid dynamics investigation within a normal artery. *J. Appl. Physiol. (1985)*. **97** (1), 424–430 (2004). discussion 416. 14766776 10.1152/japplphysiol.01329.2003

[51] Beyene, S. et al. Comparison of endothelial shear stress between ultrathin strut bioresorbable polymer drug-eluting stent vs durable-polymer drug-eluting stent post-stent implantation: An optical coherence tomography substudy from BIOFLOW II. *Cardiovasc. Revasc Med.* **61**, 26–34 (2024). 38042738 10.1016/j.carrev.2023.11.014

[52] Chiastra, C. et al. Patient-specific modeling of stented coronary arteries reconstructed from optical coherence tomography: Towards a widespread clinical use of fluid dynamics analyses. *J. Cardiovasc. Transl Res.* **11**(2), 156–172 (2018). 29282628 10.1007/s12265-017-9777-6 PMC5908818

[53] Migliori, S. et al. Application of an OCT-based 3D reconstruction framework to the hemodynamic assessment of an ulcerated coronary artery plaque. *Med. Eng. Phys.* **78**, 74–81 (2020). 32037282 10.1016/j.medengphy.2019.12.006

[54] Wu, W. et al. Three dimensional reconstruction of coronary artery stents from optical coherence tomography: experimental validation and clinical feasibility. *Sci. Rep.* **11** (1), 12252 (2021). 34112841 10.1038/s41598-021-91458-y PMC8192920

[55] Bourantas, C. V. et al. Effect of the endothelial shear stress patterns on neointimal proliferation following drug-eluting bioresorbable vascular scaffold implantation: an optical coherence tomography study. *JACC Cardiovasc. Interv*. **7** (3), 315–324 (2014). 24529931 10.1016/j.jcin.2013.05.034

[56] Beyene, S. et al. Comparison of endothelial shear stress between ultrathin strut bioresorbable polymer drug-eluting stent vs durable-polymer drug-eluting stent post-stent implantation: An optical coherence tomography substudy from BIOFLOW II. *Cardiovasc. Revascularization Med.* **61**, 26–34 (2024). 10.1016/j.carrev.2023.11.014 38042738

[57] Tenekecioglu, E. et al. Early strut protrusion and late neointima thickness in the Absorb bioresorbable scaffold: A serial wall shear stress analysis up to five years. *EuroIntervention* **15**(4), e370–e379 (2019). 29969424 10.4244/EIJ-D-18-00381

[58] Cheng, C. P., Parker, D. & Taylor, C. A. Quantification of wall shear stress in large blood vessels using Lagrangian interpolation functions with cine phase-contrast magnetic resonance imaging. *Ann. Biomed. Eng.* **30** (8), 1020–1032 (2002). 12449763 10.1114/1.1511239

[59] Fonken, J. et al. The impact of a limited field-of-view on computed hemodynamics in abdominal aortic aneurysms: evaluating the feasibility of completing ultrasound segmentations with parametric geometries. *Ann. Biomed. Eng.* **51**(6), 1296–1309 (2023). 36709232 10.1007/s10439-022-03133-6 PMC10172266

[60] Johnson, K., Sharma, P. & Oshinski, J. Coronary artery flow measurement using navigator echo gated phase contrast magnetic resonance velocity mapping at 3.0 T. *J. Biomech.* **41** (3), 595–602 (2008). 18036532 10.1016/j.jbiomech.2007.10.010 PMC2759278

[61] Pantos, I. et al. In vivo wall shear stress measurements using phase-contrast MRI. *Expert Rev. Cardiovasc. Ther.* **5** (5), 927–938 (2007). 17867922 10.1586/14779072.5.5.927

[62] Wang, I. C. et al. Wall shear stress mapping for human femoral artery based on ultrafast ultrasound vector Doppler estimations. *Med. Phys.* **48** (11), 6755–6764 (2021). 34525217 10.1002/mp.15230

[63] Xiaoyong, T. Y., Wei, C., Juan, H., Feng, C. & Zhuo, Q. L. Evaluation efficacy wall shear stress carotid artery stenting. *Heliyon* **10**(11) (2024). 10.1016/j.heliyon.2024.e31383 PMC11140617 38828314

[64] Aizawa, K. et al. Brachial artery vasodilatory response and wall shear rate determined by multigate Doppler in a healthy young cohort. *J. Appl. Physiol. (1985)*. **124** (1), 150–159 (2018). 28935823 10.1152/japplphysiol.00310.2017 PMC5866444

[65] Huang, Y. H. et al. Estimation of mouse carotid arterial wall shear stress using high-frequency ultrasound imaging. *IEEE Trans. Ultrason. Ferroelectr. Freq. Control* **70**(6), 474–485 (2023). 37015118 10.1109/TUFFC.2023.3262275

[66] Yiu, B. Y. & Yu, A. C. Least-squares multi-angle Doppler estimators for plane-wave vector flow imaging. *IEEE Trans. Ultrason. Ferroelectr. Freq. Control* **63**(11), 1733–1744 (2016). 27824557 10.1109/TUFFC.2016.2582514

[67] Lindsey, B. D. et al. 3-D intravascular characterization of blood flow velocity fields with a forward-viewing 2-D array. *Ultrasound Med. Biol.* **46**(9), 2560–2571 (2020). 32616428 10.1016/j.ultrasmedbio.2020.05.022 PMC7429285

[68] Rojas, S. S. et al. High-frequency, 2-mm-diameter forward-viewing 2-D array for 3-D Intracoronary blood flow imaging. *IEEE Trans. Ultrason. Ferroelectr. Freq. Control* **71**(8), 1051–1061 (2024). 38913530 10.1109/TUFFC.2024.3418708 PMC11381909

[69] Douglas, J. S. Jr. et al. Coronary stent restenosis in patients treated with cilostazol. *Circulation* **112** (18), 2826–2832 (2005). 16246948 10.1161/CIRCULATIONAHA.104.530097

[70] Holmes, D. R. Jr. et al. ACCF/AHA clopidogrel clinical alert: approaches to the FDA boxed warning: a report of the American College of Cardiology Foundation Task Force on clinical expert consensus documents and the American Heart Association endorsed by the Society for Cardiovascular Angiography and Interventions and the Society of Thoracic Surgeons. *J. Am. Coll. Cardiol.* **56** (4), 321–341 (2010). 20633831 10.1016/j.jacc.2010.05.013

[71] Dziopa, K. et al. Digital twins: reimagining the future of cardiovascular risk prediction and personalised care. *Hellenic J. Cardiol.* **81**, 4–8 (2025). 38852883 10.1016/j.hjc.2024.06.001

[72] Sel, K. et al. Building digital twins for cardiovascular health: From principles to clinical impact. *J. Am. Heart Assoc.* **13**(19), e031981 (2024). 39087582 10.1161/JAHA.123.031981 PMC11681439

[73] Akhtar, S. et al. CFD analysis on blood flow inside a symmetric stenosed artery: Physiology of a coronary artery disease. *Sci. Prog*. **106** (2), 368504231180092 (2023). 37292014 10.1177/00368504231180092 PMC10450296

[74] Timofeeva, M. et al. Numerical simulation of the blood flow through the coronary artery stenosis: Effects of varying eccentricity. *Comput. Biol. Med.* **146**, 105672 (2022). 35661622 10.1016/j.compbiomed.2022.105672

[75] Eslami, P. et al. Effect of wall elasticity on hemodynamics and wall shear stress in patient-specific simulations in the coronary arteries. *J. Biomech. Eng.* **142**(2), 0245031–02450310 (2020). 31074768 10.1115/1.4043722 PMC7105147

[76] Samady, H. et al. Coronary artery wall shear stress is associated with progression and transformation of atherosclerotic plaque and arterial remodeling in patients with coronary artery disease. *Circulation* **124**(7), 779–788 (2011). 21788584 10.1161/CIRCULATIONAHA.111.021824

[77] Suo, J., Oshinski, J. N. & Giddens, D. P. Blood flow patterns in the proximal human coronary arteries: Relationship to atherosclerotic plaque occurrence. *Mol. Cell. Biomech.* **5**(1), 9–18 (2008). 18524242

[78] Veneziani, A. & Vergara, C. Flow rate defective boundary conditions in haemodynamics simulations. *Int. J. Numer. Methods Fluids*. **47** (8–9), 803–816 (2005).

[79] Da Veiga, L. B. et al. The virtual element method. *Acta Numerica*. **32**, 123–202 (2023).

[80] da Veiga, L. B., Mora, D. & Vacca, G. The stokes complex for virtual elements with application to navier-stokes flows. *J. Sci. Comput.* **81**(2), 990–1018 (2019).

[81] Jin, X. W. et al. NSFnets (Navier-Stokes flow nets): Physics-informed neural networks for the incompressible Navier-Stokes equations.. *J. Comput. Phys.*10.1016/j.jcp.2020.109951 (2021).

[82] Oldenburg, J. et al. Geometry aware physics informed neural network surrogate for solving Navier-Stokes equation (GAPINN).. *Adv. Model. Simul. Eng. Sci.*10.1186/s40323-022-00221-z (2022).

[83] Taylor, C. A. & Figueroa, C. A. Patient-specific modeling of cardiovascular mechanics. *Annu. Rev. Biomed. Eng.* **11**, 109–134 (2009). 19400706 10.1146/annurev.bioeng.10.061807.160521 PMC4581431

[84] Formaggia, L., Quarteroni, A. & Veneziani, A. *Cardiovascular Mathematics: Modeling and simulation of the circulatory system* Vol. 1 (Springer Science & Business Media, 2010).

[85] Formaggia, L., Lamponi, D. & Quarteroni, A. One-dimensional models for blood flow in arteries. *J. Eng. Math.* **47** (3–4), 251–276 (2003).

[86] Chiastra, C. et al. On the necessity of modelling fluid-structure interaction for stented coronary arteries. *J. Mech. Behav. Biomed. Mater.* **34**, 217–230 (2014). 24607760 10.1016/j.jmbbm.2014.02.009

[87] Zeng, D. et al. Effects of cardiac motion on right coronary artery hemodynamics. *Ann. Biomed. Eng.* **31** (4), 420–429 (2003). 12723683 10.1114/1.1560631

[88] Kim, S. et al. Dynamic coronary blood flow velocity and wall shear stress estimation using ultrasound in an ex vivo porcine heart. *Cardiovasc. Eng. Technol.* **15**(1), 65–76 (2024). 37962814 10.1007/s13239-023-00697-9 PMC10923141

[89] Tierney, J. et al. Adaptive clutter demodulation for non-contrast ultrasound perfusion imaging. *IEEE Trans. Med. Imaging* **36**(9), 1979–1991 (2017). 28622670 10.1109/TMI.2017.2714901 PMC5605932

[90] Tierney, J. et al. Independent component-based spatiotemporal clutter filtering for slow flow ultrasound. *IEEE Trans. Med. Imaging* **39**(5), 1472–1482 (2020). 31689187 10.1109/TMI.2019.2951465 PMC7288756

[91] Ozgun, K. A. & Byram, B. C. Multidimensional clutter filtering of aperture domain data for improved blood flow sensitivity. *IEEE Trans. Ultrason. Ferroelectr. Freq. Control* **68**(8), 2645–2656 (2021). 33852387 10.1109/TUFFC.2021.3073292 PMC8345228

[92] Kripfgans, O. D. et al. Three-dimensional US for quantification of volumetric blood flow: multisite multisystem results from within the quantitative imaging biomarkers alliance. *Radiology* **296**(3), 662–670 (2020). 32602826 10.1148/radiol.2020191332 PMC7457950

[93] Pinter, S. Z. et al. Color flow ultrasound spatial sampling beam density for partial volume-corrected three-dimensional volume flow (3DVF): Theory, simulation, and experiment. *Ultrasound Med. Biol.* **50**(8), 1122–1133 (2024). 38729810 10.1016/j.ultrasmedbio.2024.03.015 PMC13455765

[94] Welsh, A. W. et al. Three-dimensional US fractional moving blood volume: Validation of renal perfusion quantification. *Radiology* **293**(2), 460–468 (2019). 31573404 10.1148/radiol.2019190248 PMC6800606

[95] Lefieux, A. et al. Semi-automatic reconstruction of patient-specific stented coronaries based on data assimilation and computer aided design. *Cardiovasc. Eng. Technol.* **13**(4), 517–534 (2022). 34993928 10.1007/s13239-021-00570-7

[96] Nannini, G. et al. An automated and time-efficient framework for simulation of coronary blood flow under steady and pulsatile conditions. *Comput. Methods Programs Biomed.* **257**, 108415 (2024). 39270532 10.1016/j.cmpb.2024.108415

[97] Dodge, J. T. Jr. et al. Lumen diameter of normal human coronary arteries. Influence of age, sex, anatomic variation, and left ventricular hypertrophy or dilation. *Circulation* **86** (1), 232–246 (1992). 1535570 10.1161/01.cir.86.1.232

[98] Adjedj, J. et al. Visual and quantitative assessment of coronary stenoses at angiography versus fractional flow reserve: The impact of risk factors.. *Circ. Cardiovasc. Imaging*10.1161/CIRCIMAGING.117.006243 (2017). 28687539 10.1161/CIRCIMAGING.117.006243

[99] Harris, P. J. et al. The prognostic significance of 50% coronary stenosis in medically treated patients with coronary artery disease. *Circulation* **62** (2), 240–248 (1980). 7397965 10.1161/01.cir.62.2.240

[100] Yadalam, P. K. et al. Advanced machine learning for estimating vascular occlusion percentage in patients with ischemic heart disease and periodontitis. *Int. J. Cardiol. Cardiovasc. Risk Prev.* **21**, 200291 (2024). 39118994 10.1016/j.ijcrp.2024.200291 PMC11305989

[101] Rosenthal, R. L. The 50% coronary stenosis. *Am. J. Cardiol.* **115** (8), 1162–1165 (2015). 25726382 10.1016/j.amjcard.2015.01.553

[102] Tonino, P. A. et al. Angiographic versus functional severity of coronary artery stenoses in the FAME study fractional flow reserve versus angiography in multivessel evaluation. *J. Am. Coll. Cardiol.* **55** (25), 2816–2821 (2010). 20579537 10.1016/j.jacc.2009.11.096

[103] Madsen, E. L. et al. Tissue mimicking materials for ultrasound phantoms. *Med. Phys.* **5** (5), 391–394 (1978). 713972 10.1118/1.594483

[104] Telichko, A. V., Dahl, J. J. & Herickhoff, C. D. Cylindrical transducer array for intravascular shear wave elasticity imaging: Preliminary development. *IEEE Trans. Ultrason. Ferroelectr. Freq. Control* **69**(3), 1077–1087 (2022). 34990357 10.1109/TUFFC.2022.3140976

[105] Neumann, F. J. et al. 2018 ESC/EACTS Guidelines on myocardial revascularization. *Eur. Heart J.* **40** (2), 87–165 (2019). 30165437 10.1093/eurheartj/ehy394

[106] Ng, J. C. K. et al. Stent malapposition generates stent thrombosis: Insights from a thrombosis model. *Int. J. Cardiol.* **353**, 43–45 (2022). 35143874 10.1016/j.ijcard.2022.02.003

[107] Lindsey, B. D. et al. Acoustic characterization of contrast-to-tissue ratio and axial resolution for dual-frequency contrast-specific acoustic angiography imaging. *IEEE Trans. Ultrason. Ferroelectr. Freq. Control*. **61** (10), 1668–1687 (2014). 25265176 10.1109/TUFFC.2014.006466 PMC8375273

[108] de Bruyne, B. et al. Simultaneous coronary pressure and flow velocity measurements in humans. Feasibility, reproducibility, and hemodynamic dependence of coronary flow velocity reserve, hyperemic flow versus pressure slope index, and fractional flow reserve. *Circulation* **94** (8), 1842–1849 (1996). 8873658 10.1161/01.cir.94.8.1842

[109] Hozumi, T. et al. Noninvasive assessment of coronary flow velocity and coronary flow velocity reserve in the left anterior descending coronary artery by Doppler echocardiography: comparison with invasive technique. *J. Am. Coll. Cardiol.* **32** (5), 1251–1259 (1998). 9809933 10.1016/s0735-1097(98)00389-1

[110] Kumar, A. et al. High coronary shear stress in patients with coronary artery disease predicts myocardial infarction. *J. Am. Coll. Cardiol.* **72**(16), 1926–1935 (2018). 30309470 10.1016/j.jacc.2018.07.075

[111] Kumar, A. et al. Low coronary wall shear stress is associated with severe endothelial dysfunction in patients with nonobstructive coronary artery disease. *JACC: Cardiovasc. Interventions*. **11** (20), 2072–2080 (2018). 10.1016/j.jcin.2018.07.004 PMC6217963 30268874

[112] Tufaro, V. et al. Can fast wall shear stress computation predict adverse cardiac events in patients with intermediate non-flow limiting stenoses? *Atherosclerosis* **401**, 119099 (2025). 39813850 10.1016/j.atherosclerosis.2024.119099

[113] Demene, C. et al. Spatiotemporal clutter filtering of Ultrafast ultrasound data highly increases Doppler and fUltrasound sensitivity. *IEEE Trans. Med. Imaging* **34**(11), 2271–2285 (2015). 25955583 10.1109/TMI.2015.2428634

[114] Jensen, J., Stuart, M. B. & Jensen, J. A. High frame rate vector velocity estimation using plane waves and transverse oscillation. In *IEEE International Ultrasonics Symposium* (IUS), 1–4 (2015).

[115] Jing, B. et al. A transverse velocity spectral estimation method for ultrafast ultrasound Doppler imaging. *IEEE Trans. Ultrason. Ferroelectr. Freq. Control* **70**(12), 1749–1760 (2023). 37721880 10.1109/TUFFC.2023.3316748 PMC10762297

[116] Pihl, M. J. & Jensen, J. A. A transverse oscillation approach for estimation of three-dimensional velocity vectors, part I: concept and simulation study. *IEEE Trans. Ultrason. Ferroelectr. Freq. Control*. **61** (10), 1599–1607 (2014). 25265170 10.1109/TUFFC.2013.006237

[117] Cignoni, P. et al. Meshlab: an open-source mesh processing tool. In *Eurographics Italian chapter conference* (Salerno, 2008).

[118] Autodesk About Autodesk^®^ Netfabb^®^ (2022).

[119] Izzo, R. et al. The vascular modeling toolkit: a python library for the analysis of tubular structures in medical images. *J. Open. Source Softw.* **3** (25), 745 (2018).

[120] Heywood, J. G., Rannacher, R. & Turek, S. Artificial boundaries and flux and pressure conditions for the incompressible Navier–Stokes equations. *Int. J. Numer. Methods Fluids*. **22** (5), 325–352 (1996).

[121] Myronenko, A. & Song, X. Point set registration: Coherent point drift. *IEEE Trans. Pattern Anal. Mach. Intell.* **32**(12), 2262–2275 (2010). 20975122 10.1109/TPAMI.2010.46

[122] Zhang, J., Yao, Y. & Deng, B. Fast and robust iterative closest point. *IEEE Trans. Pattern Anal. Mach. Intell.* **44**(7), 3450–3466 (2022). 33497327 10.1109/TPAMI.2021.3054619

