# Supplementary material for: Intravascular ultrasound wall shear stress imaging in stented coronary arteries with ultrafast Doppler
Source: Sci Rep. 2026 Apr 9;16:16201. doi: 10.1038/s41598-026-47719-9 (PMC13201558; doi:10.1038/s41598-026-47719-9)
Supplement: Supplementary file 1 — Supplementary Material 1 [file 41598_2026_47719_MOESM1_ESM.pdf]

SUPPLEMENTARY INFORMATION

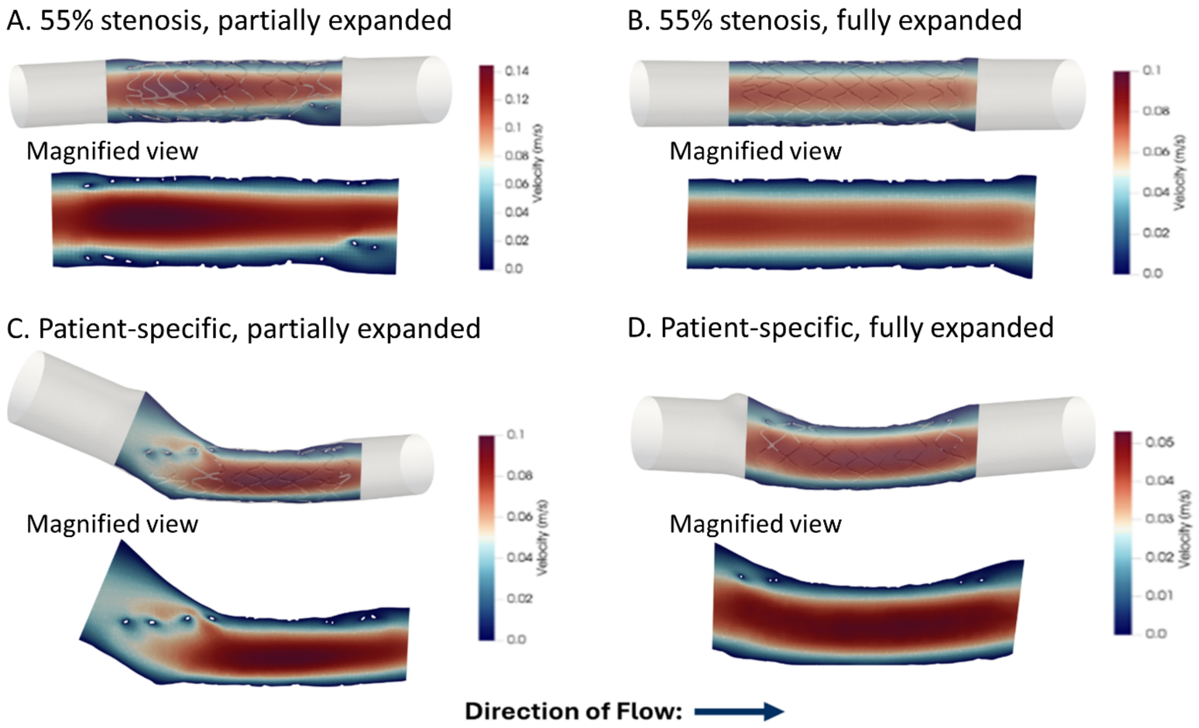

Fig. S1. CFD velocity contours on a cross-sectional slice located along the length of each vessel for: (A) 55% stenosis with a partially expanded stent, (B) 55% stenosis with a fully-expanded stent, (C) Patient-specific vessel geometry with a partially expanded stent, and (D) Patient-specific vessel geometry with a fully expanded stent. The direction of flow is from left to right.

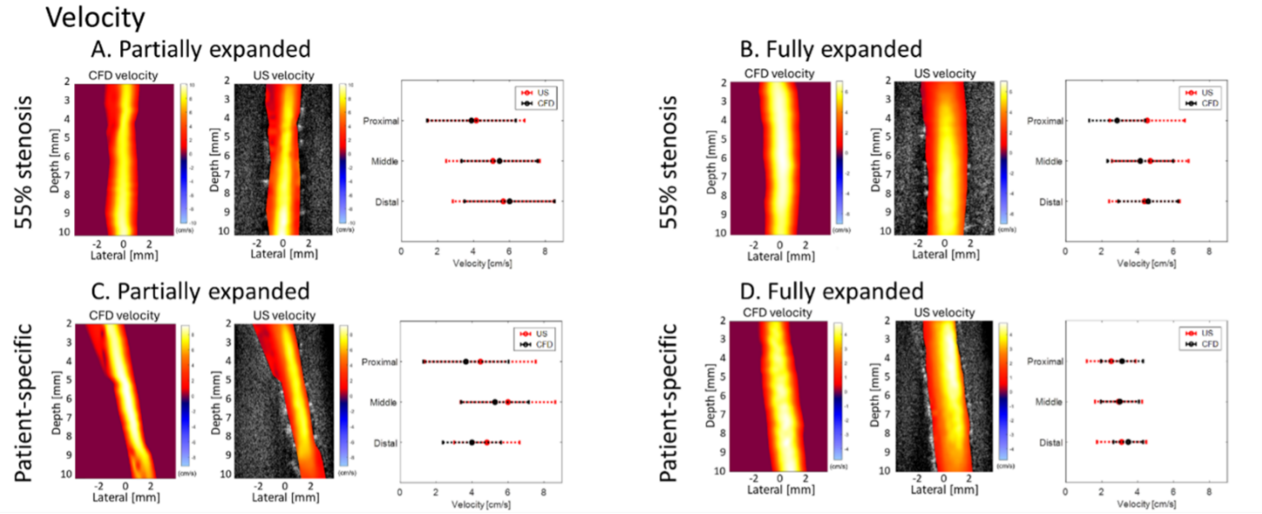

Fig. S2. CFD and ultrasound-derived velocity maps for (A) a partially expanded stent in a 55% stenosis over 4 mm, and (B) the same geometry after the stent is fully expanded. In the bottom row, the ultrasound and CFD derived WSS maps are shown for (C) a partially expanded stent in a patient-specific geometry, and (D) the same geometry after the stent is fully expanded. The mean and standard deviation of values for each segment are shown on the right side.
